# Energy-Efficient Edge and Cloud Image Classification with Multi-Reservoir Echo State Network and Data Processing Units

**DOI:** 10.3390/s24113640

**Published:** 2024-06-04

**Authors:** E. J. López-Ortiz, M. Perea-Trigo, L. M. Soria-Morillo, J. A. Álvarez-García, J. J. Vegas-Olmos

**Affiliations:** 1 Department of Computer Science and Artificial Intelligence, Universidad de Sevilla, Avda. Reina Mercedes, s/n, 41004 Sevilla, Spain; elortiz@us.es; 2 Department of Languages and Computer Systems, Universidad de Sevilla, Avda. Reina Mercedes, s/n, 41004 Sevilla, Spain; mptrigo@us.es (M.P.-T.); lsoria@us.es (L.M.S.-M.); 3 NVIDIA Corporation, Hermon Building, Yokneam 6121002, Israel; juanj@nvidia.com

**Keywords:** state network, energy-efficient models, image classification, reservoir computing, CloudCast

## Abstract

In an era dominated by Internet of Things (IoT) devices, software-as-a-service (SaaS) platforms, and rapid advances in cloud and edge computing, the demand for efficient and lightweight models suitable for resource-constrained devices such as data processing units (DPUs) has surged. Traditional deep learning models, such as convolutional neural networks (CNNs), pose significant computational and memory challenges, limiting their use in resource-constrained environments. Echo State Networks (ESNs), based on reservoir computing principles, offer a promising alternative with reduced computational complexity and shorter training times. This study explores the applicability of ESN-based architectures in image classification and weather forecasting tasks, using benchmarks such as the MNIST, FashionMnist, and CloudCast datasets. Through comprehensive evaluations, the Multi-Reservoir ESN (MRESN) architecture emerges as a standout performer, demonstrating its potential for deployment on DPUs or home stations. In exploiting the dynamic adaptability of MRESN to changing input signals, such as weather forecasts, continuous on-device training becomes feasible, eliminating the need for static pre-trained models. Our results highlight the importance of lightweight models such as MRESN in cloud and edge computing applications where efficiency and sustainability are paramount. This study contributes to the advancement of efficient computing practices by providing novel insights into the performance and versatility of MRESN architectures. By facilitating the adoption of lightweight models in resource-constrained environments, our research provides a viable alternative for improved efficiency and scalability in modern computing paradigms.

## 1. Introduction

In the dynamic technology landscape of today, the remarkable growth of software as a service (SaaS) has been accelerated by rapid advances in cloud computing and edge computing. There is an increasing demand for efficient and lightweight models that can be deployed on resource-constrained devices such as data processing units (DPUs [1]) or home stations. DPUs have emerged as promising components in cloud and edge computing architectures, providing high-performance computing capabilities while minimizing latency and power consumption. These devices are typically installed in servers or server farms and remain continuously active, making them ideal candidates for implementing models that can be trained during their idle time. However, because they are resource-constrained devices that do not have a graphical processing unit (GPU), it is essential to develop models that are optimized for these devices to fully exploit the potential of DPUs.

Meanwhile, recent years have seen unprecedented growth in the use of artificial intelligence-based models, with the vast majority of companies adopting these techniques, particularly in the areas of speech recognition and image classification. Among these applications, image classification plays a key role in computer vision, with wide-ranging applications in object recognition, autonomous vehicles, and medical imaging. However, traditional deep learning models, such as convolutional neural networks (CNNs), tend to have significant computational and memory requirements. This makes them energetically inefficient models and also complicates their training in resource-limited environments. For instance, a simple feed-forward neural network with just two hidden layers of 128 units each may have a total of around 100,000 trainable parameters (weights and biases). More complex architectures, such as deep neural networks (DNNs), can have much larger parameter sets, sometimes in the hundreds of thousands or even millions. This complexity poses significant hurdles when attempting to deploy these networks on compact devices operating at the transport layer, such as DPUs.

An overview of some commonly used CNN architectures and their respective number of trainable parameters is given in Table 1.

In 1991, Kirby presented the foundations of reservoir computing (RC) [2]. This popular approach to machine learning utilities the dynamics of a high-dimensional dynamical system, known as a reservoir, to process and classify input signals. The concepts of RC laid the groundwork for Echo State Networks (ESNs), introduced by Jaeger in 2001 [3], which have gained widespread recognition for their remarkable effectiveness in time series analysis.

One of the principal advantages of this architecture is that the network weights are determined through linear regression, a computationally more efficient method than gradient descent. Additionally, ESNs generally require fewer trainable parameters compared to deep neural networks (DNNs). Together, these factors result in significantly shorter training times for ESNs compared to DNNs.

The objectives of this study were several. Firstly, we aimed to extend the experiments conducted in our previous work [4], where we investigated the applicability of ESN-based architectures to image classification tasks beyond their conventional use in time series analysis. To achieve this, we focused on identifying the most efficient architecture for this task, which we have identified as the grouped ESN-based architecture [5].

For clarity, we will refer to the modification of this architecture for classification tasks as Multi-Reservoir ESN (MRESN). Subsequently, we explored the application of the MRESN architecture in scenarios characterized by both spatial and temporal relationships. Utilizing the CloudCast dataset [6], we employed the network to predict cloud coverage patterns over specific regions. Finally, we conducted a comparative performance analysis of the MRESN model on three resource-constrained devices—a DPU, a Raspberry Pi 4, and a Raspberry Pi 5—to assess its suitability for various computing environments.

The MRESN architecture combines the power of reservoir computing to design compact and lightweight models suitable for deployment on devices with limited resources. The fast training of MRESNs allows for continuous training, eliminating the reliance on static, pre-trained models used solely for inference. The dynamic adaptability of MRESN to changing input signals, such as weather forecasts, through continuous training, is perfectly suited to the requirements of low-resource devices, where lightweight, flexible models are paramount.

In the following sections, we dive into the state-of-the-art models, where we will look at some previously proposed ESN-based architectures. We then present a modification to these architectures, adapting them to an image-based classification task. Additionally, we discuss the experimental setup, covering training and evaluation procedures, and present the results obtained from our experiments on the MNIST, FashionMNIST, and CloudCast datasets. Finally, we discuss the implications of our findings, emphasizing the potential of this kind of architectures for cloud computing and edge computing applications where lightweight models are crucial.

In conclusion, our study makes a valuable contribution to the ongoing research in efficient and lightweight ESN-based models for low-resource devices. These findings emphasize the potential of MRESN for practical implementation in resource-constrained environments, marking a significant step forward in the field of edge computing and efficient model deployment.

## 2. Previous Works

Echo State Networks (ESNs) were first introduced in the early 2000s [3] as a type of recurrent neural network. The original motivation for ESNs was to overcome the problems associated with training traditional RNNs, which have recurrent connections that make backpropagation difficult for long sequences.

Within the realm of neural networks, deep neural networks (DNNs) and Echo State Networks (ESNs) constitute two distinct paradigms for learning. DNNs entail the construction of intricate networks with multiple interconnected layers of neurons, fine-tuned through backpropagation to adapt the connection weights and enhance predictive accuracy. In contrast, ESNs are characterized by three fundamental components: an input layer, a reservoir layer comprising neurons arranged in a graph-like structure, and an output layer. Both the input and reservoir layers are randomly generated, maintaining fixed weights throughout training. In ESNs, the emphasis lies in the quest for the optimal weight set within the output layer to facilitate precise predictions on input data. Furthermore, these weight adjustments are achieved through a computationally less intensive linear regression algorithm, as opposed to the resource-intensive backpropagation methods employed in DNNs. In summary, while DNNs aim to iteratively fine-tune all network weights throughout training, ESNs leverage a fixed, random structure to make predictions, contingent upon an optimized output layer.

In exclusively training the output layer weights, ESNs offer a notable advantage over conventional DNNs. This advantage manifests in reduced training duration, diminished computational requirements, and decreased reliance on extensive training datasets. The simplicity and robustness inherent in ESNs, combined with their efficient training, minimal resource consumption, and proficiency in approximating intricate system dynamics, make them an enticing choice for researchers across diverse fields. ESNs have found successful application in various domains, spanning natural language processing [7,8], speech recognition [9], the prediction of polymerizing process [10], image classification [11], bioinformatics [12], control systems [13], and music generation [14].

ESNs have further demonstrated their effectiveness in image processing, as exemplified by Schaetti et al. [15] and Barredo et al. [16], who transformed images into temporal sequences and processed them using ESNs. In [17], the potential of reservoir computing in image recognition was demonstrated using a reservoir computing network (RCN). Tong et al. [18] discussed that achieving high performance in image recognition with raw image data requires a large-scale reservoir with a substantial number of neurons. To overcome this bottleneck, they proposed a method that combines reservoir computing with untrained convolutional neural networks. Sun et al. [11] compared ESNs to LSTMs, showing the potential of reservoir computing. In [19], an image array was passed through a noise filter multiple times as the ESN converged to a classification. Except for [17], all works have tested ESNs using the MNIST dataset. A comprehensive overview of these studies and their respective scopes is provided in Table 2.

ESNs have proven to be versatile networks, performing well in a variety of tasks. This has generated interest in exploring architectures composed of multiple reservoirs [5,23].

In some cases, ESNs have been combined with other types of neural networks to improve their performance. For example, Souahlia et al. [20] combined an ESN with a Multi-Layer Perceptron (MLP) for color image segmentation, and Yang et al. [21] used a combination of Long Short-Term Memory (LSTM) and ESNs for temperature control in an industrial hot-blast furnace. The integration of ESNs with convolutional neural networks (CNNs) has also been explored, as shown in the study by Mustaqeem et al. [22], where ESNs were used in a solar energy prediction task. In addition, some modifications to the original architecture have been tested with good results [21,24].

On the other hand, data processing units (DPUs) are specialized network interface cards (NICs) engineered to offload processing tasks traditionally handled by the system CPU. DPUs come equipped with dedicated processors optimized for tasks like encryption/decryption, firewall functions, and network processing. This makes DPUs particularly well suited for deployment in high-traffic web servers, where they effectively relieve the CPU of processing burdens, resulting in improved overall system performance.

Recent research has demonstrated the capability of DPUs to offload critical workloads and enhance system performance. In a comprehensive study in a 100 Gbps Ethernet environment, Liu et al. [25] compared DPU and server performance. While the DPU did not outperform the server in tasks like intrusion detection and network address translation, it excelled in encryption/decryption, memory operations with heavy contention, and interprocess communication.

In another study [26], researchers successfully offloaded IPsec encryption to DPUs’ hardware accelerators, resulting in substantial performance gains compared to workstation CPU-based encryption. This approach promises improved network performance and enhanced security for Ethernet traffic.

Barsellotti et al. [1] explored innovative use cases for DPUs in edge scenarios, particularly in integrating AI-based operations directly into the network for tasks like intrusion detection. DPUs proved effective in native integration, offering performance and network intelligence advantages.

In the realm of deep learning, DPUs have found application in various stages of model training, including data augmentation and validation. Leveraging DPUs for these tasks, Jain et al. [27] achieved up to a 15% increase in training performance. Their subsequent work [28] demonstrated consistent performance improvements for CNNs and Transformer models, both in weak and strong scaling scenarios across multiple nodes.

In the following section, we introduce a modification to ESN-based networks that enables their use in image classification tasks. This modification involves replacing the final layer with a specialized output layer with multiple outputs, each trained to discriminate whether an image belongs to a particular class. Additionally, we incorporated a final argmax layer, which processes the outputs from this layer to determine the class with the highest probability, thus facilitating classification.

## 3. ESN Architectures

ESNs have gained significant attention in recent years due to their effectiveness in modeling complex temporal dependencies in various applications, including time series prediction, signal processing, and pattern recognition. In this section, we delve into the diverse architectures and variants of ESNs that have been proposed to enhance their performance and applicability across different domains. We begin by exploring the foundational principles of ESNs, elucidating their core components and operational mechanisms. Subsequently, we delve into the evolution of ESN architectures, tracing their development from basic reservoir networks to more sophisticated variants tailored to specific tasks and challenges.

### 3.1. Vanilla-ESN

The main features of basic ESN architecture can be summarized in the following key points:

- Reservoir (*W*): The reservoir is the core component of an ESN and consists of a large number of recurrently connected neurons. These connections are randomly initialized and remain fixed throughout the training process. The reservoir neurons receive input signals from the input layer and also receive feedback from other neurons within the reservoir.
- Input Layer ($W_{in}$): The input layer of an ESN receives external input signals or features. Each input signal is connected to all neurons in the reservoir with corresponding input weights. The input signals can be scalars, vectors, or even time series data.
- Reservoir Dynamics: The dynamics of the reservoir neurons play a crucial role in processing input signals. The internal state of the reservoir evolves over time as it receives input signals and recurrent feedback from other neurons. This dynamic behavior allows the network to capture temporal dependencies in the input data.
- Output Layer ($W_{out}$): The output layer of an ESN is typically a linear readout layer that receives the activations of the reservoir neurons as input. The output layer computes the network’s output by applying a set of output weights to the reservoir activations. The output can be a scalar, vector, or time series, depending on the task.
- Training: Unlike traditional recurrent neural networks (RNNs), where all network parameters are trained using backpropagation through time (BPTT), ESNs employ a simple and efficient training strategy. Only the output weights connecting the reservoir to the output layer are trained using linear regression or a similar method. This training step is fast and computationally efficient since it involves solving a linear system of equations.
- Echo State Property: One of the key properties of ESNs is the echo state property, which ensures that the dynamics of the reservoir exhibit a rich repertoire of transient behaviors. This property is essential for capturing complex temporal patterns in the input data. The echo state property is closely related to one of the most important hyperparameters in ESNs, the spectral radius (ρ). The spectral radius of an ESN is a crucial hyperparameter that influences the network’s dynamics and performance. It represents the maximum absolute eigenvalue of the reservoir weight matrix. Essentially, it determines the scaling of the recurrent connections within the reservoir. A larger spectral radius implies stronger internal dynamics and a potentially richer representation of input data in the reservoir. However, if the spectral radius is too large, the reservoir may become chaotic, leading to unstable behavior and difficulty in training. Conversely, a smaller spectral radius can lead to vanishing gradients and poor performance, as the dynamics within the reservoir may become too weak to capture meaningful patterns in the input data. Choosing an appropriate spectral radius is essential for achieving good performance in ESNs. It often involves experimentation and tuning based on the specific task and dataset.
- Reservoir Initialization: The random initialization of the reservoir connections is crucial for ensuring the echo state property. The spectral radius of the reservoir weight matrix, which measures the largest eigenvalue, is often controlled to regulate the network’s dynamics and stability.

Overall, the basic architecture of an ESN consists of a reservoir of recurrently connected neurons, an input layer for receiving external input signals, and an output layer for computing the network’s output. The reservoir’s dynamics, combined with the trainable output weights, enable ESNs to effectively model and predict temporal sequences in various applications.

The first step in ESN training is a random initialization of both the reservoir and the input layer. Once initialized, the network enters the training phase where it learns to map the input patterns to the desired output patterns. This is achieved by presenting the network with input sequences and storing changes in the reservoir state in a matrix, which we will call *H* for ease of reading. This matrix is then used to adjust the output weights using a simple linear regression or optimization algorithm.

**Figure 1:**
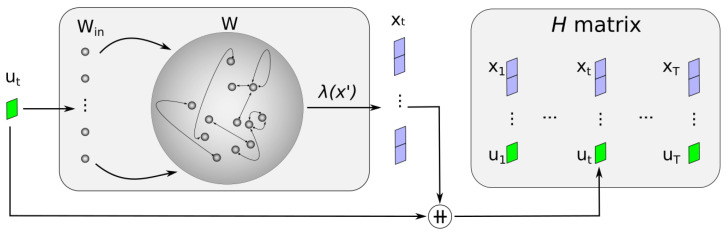
ESN training. The symbol ++ is used to express a vector concatenation. λ is used to express the computation of the new state. The length of training is expressed by T.

Figure 1 shows the training of an ESN. Each new reservoir state is associated with the input that caused it, both stored in the matrix H. We represent the function used to compute this state with λ. In general, the function for updating the state was the one proposed by Jaeger [29]. See Equation (1).

(1) $$x_{t}=\left(1-\alpha \right)x_{t-1}+\alpha S\left(W_{in}\cdot u_{t}+W\cdot x_{t-1}\right)$$

In the given equation, $W_{in}\in R^{N\times K}$ denotes the weights of the input layer, while $W\in R^{N\times N}$ is the adjacency matrix that defines the connections between neurons. These matrices play a pivotal role in transforming the input into the internal dynamics of the network. The input at time *t*, denoted by the vector $u_{t}\in R^{K}$, undergoes processing through these weights. The reservoir comprises *N* neurons with states that evolve over time. The state of these neurons at the previous time step is denoted by $x_{t-1}\in R^{N}$. Typically, the function *S* is selected to be a sigmoid function, with the hyperbolic tangent being a common choice.

This function is known as the leaking integration approach because at each step, there is a leakage of information from the previous state ($x_{t-1}$) to the new state ($x_{t}$). The amount of leaked information is controlled by the parameter α, which is known as the leaking rate, and its values are in the interval [0, 1]. The leaking rate is another of the main hyperparameters in ESN training.

The final training step is to use the H-matrix data to find optimal weights for the output layer. Linear regression with Tikhonov regularization (Equation (2)) is typically used for this purpose.

(2) $$W_{out}={\left(H^{T}H+\beta I\right)}^{-1}H^{T}Y$$

In this equation, $W_{out}$ represents the output layer weights, *H* denotes the storage matrix, *Y* is used to express the expected output, and β corresponds to the Tikhonov regularization factor. The Tikhonov regularization factor is another relevant hyperparameter in ESN training and is used to avoid overfitting.

**Figure 2:**
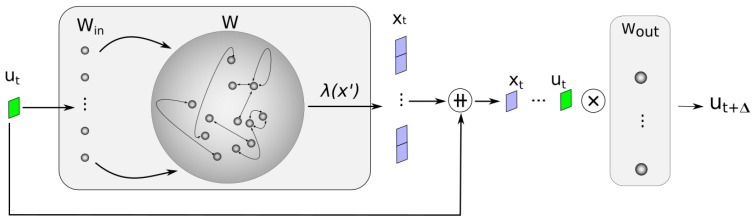
ESN Test phase. The output layer processes the new input beside the state it produces in the network to obtain the predicted output. Symbol ++ is used to express vector concatenation, while ⨂ is used to express matrix multiplication.

Once the weights of the output layer have been calculated, the network is ready to make inferences. At this stage, the new input data, together with the response they cause in the reservoir state, are processed by the output layer to make a prediction (Figure 2).

### 3.2. ESN Architecture for Classification

As proposed by [30], an alternative approach for vanilla ESN is to have an output layer with specific outputs for each class instead of relying on a single output to determine the class to which an image belongs. This approach involves designing a single output layer with multiple outputs. This output layer, called $W_{outs}$ for simplicity, will produce a vector of size 1xk, where *k* is the number of classes in the dataset. Each position in this vector will contain the probability that the input belongs to each respective class. During training, the target vector for each input image is constructed based on the class it belongs to. In employing this method, the network can learn distinct decision boundaries for different classes, potentially improving the classification accuracy. Therefore, if we take into account *k* classes, Formula (2) becomes

(3) $$W_{outs}^{c}={\left(H^{T}H+\beta I\right)}^{-1}H^{T}Y^{c},\;\;\;c\in \left[1,2,\ldots ,k\right]$$

**Figure 3:**
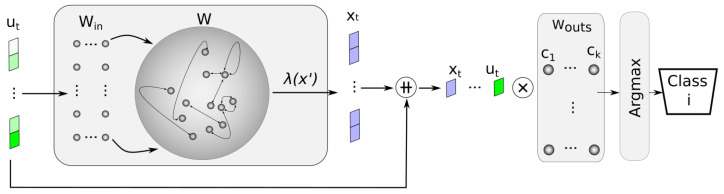
ESN with specialized output layers. Argmax or softmax could be used as the final step. Symbol ++ is used to express vector concatenation, while ⨂ is used to express matrix multiplication.

This approach does not require multiple passes through the dataset or significantly prolongs the training process, despite increasing the number of outputs in the output layer (*k*). To train this specialized output layer, we utilize the states stored in the *H* matrix and compare them with different target vectors derived from the original labels. The new target vectors are binary, containing a value of 1 when the example belongs to the class being trained, and 0 otherwise. This process allows us to develop a specialized output layer with multiple outputs that can determine the probability of an input belonging to each class. Figure 3 shows this kind of network.

In addition to the variations in the output layer compared to the basic architecture (Figure 2), we also observed a modification in the input layer, as shown in Figure 3. While the ESN architecture can be designed to accept a single data point as input, it is also possible to utilize a vector as input data. Consequently, the input layer must be structured as a matrix to accommodate inputs of varying sizes, aligning with the dimensions of the network.

### 3.3. ESN-Based Architectures for Image Classification

This capacity to process vector inputs and generate specialized output layers enables the utilization of ESNs for image processing tasks. In our prior research [4], we investigated this modification in both the vanilla ESN architecture and the architectures proposed by Gallicchio [5]. Through this exploration, we examined the behaviors of these networks in image classification tasks using the widely recognized MNIST and FashionMNIST datasets. While the achieved results may not rival those obtained from specialized techniques such as convolutional neural networks, they still warrant further investigation into the behaviors of ESN-based architectures. Two noteworthy points emerged from our analysis: firstly, the minimal impact of the alpha and rho parameters on the image classification task; and secondly, the possibility of being trained on CPUs in reasonable times, which allows their deployment on smaller devices.

**Figure 4:**
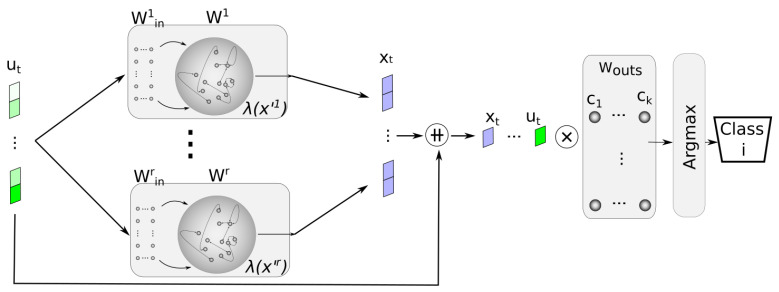
MRESN architecture. Test phase. Symbol ++ is used to express vector concatenation, while ⨂ is used to express matrix multiplication.

Among the architectures tested, the one based on the grouped ESN architecture stood out for its ability to reduce memory requirements while achieving a slightly higher accuracy rate compared to the other variants. In this architecture, known as a Multi-Reservoir Echo State Network (MRESN) for ease of understanding, multiple reservoirs operate in parallel, independently processing the input data. Each reservoir generates a state in response to the input, which varies due to the inherent randomness in reservoir generation. These states are concatenated and combined with the input data to populate the matrix *H*, which is crucial for computing the output layer weights. This approach enhances the network’s capacity to capture diverse features and patterns in the input data. Figure 4 illustrates this architectural concept.

In the following section, we build upon our exploration of the MRESN architecture in two distinct ways. Firstly, we assess the feasibility of deploying these networks on resource-constrained devices. Secondly, we expand upon the experiments conducted in our prior research utilizing the network in an image classification task with a temporal component, such as satellite image classification.

## 4. Methodology

This section includes a description of the datasets employed to evaluate the performance of our models, as well as details about the hardware infrastructure utilized to execute the experiments.

### 4.1. Hardware

Three resource-constrained devices were used in this work:

- Nvidia Mellanox BlueField-2 DPU: (Nvidia Corporation, Sunnyvale, CA, USA) These cards have been previously validated in state-of-the-art models and have demonstrated efficacy in fields such as task offloading, isolation, and acceleration for security, networking, and storage [25,27,31].
- Raspberry Pi 4: (Raspberry Pi Foundation, Cambridge, UK) This device and its predecessor and later models are widely used in the Internet of Things (IoT) and various home projects.
- Raspberry Pi 5.

Table 3 summaries their characteristics and those of the server used for the power tests.

### 4.2. MNIST and FashionMNIST Datasets

**Figure 5:**
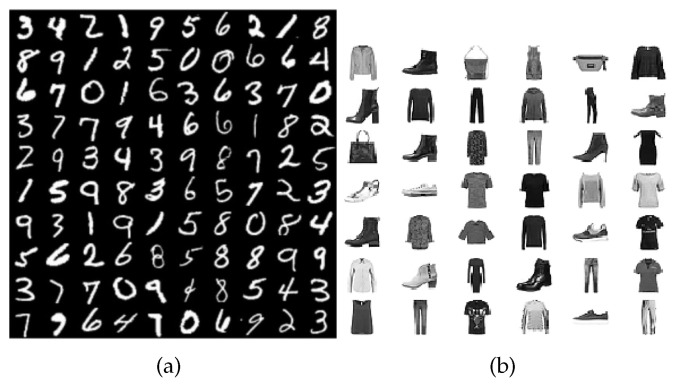
(**a**) MNIST, (**b**) FashionMNIST.

The MNIST and FashionMNIST datasets are both commonly used benchmarks for image classification. Both datasets contain training and test sets, and each example in the sets is associated with a label from a given number of classes. However, while MNIST contains ten classes of digits, FashionMNIST contains ten classes of garments, making its images more difficult to classify than MNIST (See Figure 5).

### 4.3. CloudCast Dataset

The CloudCast dataset [6] comprises satellite images utilized for cloud prediction and analysis, commonly employed in machine learning and remote sensing research to develop algorithms and models for forecasting cloud cover and weather patterns. The dataset consists of a large number of satellite images captured at various times and locations, accompanied by corresponding labels or annotations indicating different cloud types.

Containing 70,080 images with 11 different cloud types spanning multiple layers of the atmosphere, the dataset is meticulously annotated at the pixel level. In this dataset, each pixel can contain a value between 0 and 14, indicating the class to which the pixel belongs at that time. These values correspond to the following classes:

0. Cloud-free land;
1. Cloud-free sea;
2. Snow over land;
3. Sea ice;
4. No clouds or missing data;
5. Very loud clouds;
6. Low clouds;
7. Mid-level clouds;
8. High opaque clouds;
9. Very high opaque clouds;
10. Fractional clouds;
11. High semitransparent thin clouds;
12. High semitransparent moderately thick clouds;
13. High semitransparent thick clouds;
14. High semitransparent above low or medium clouds.

**Figure 6:**
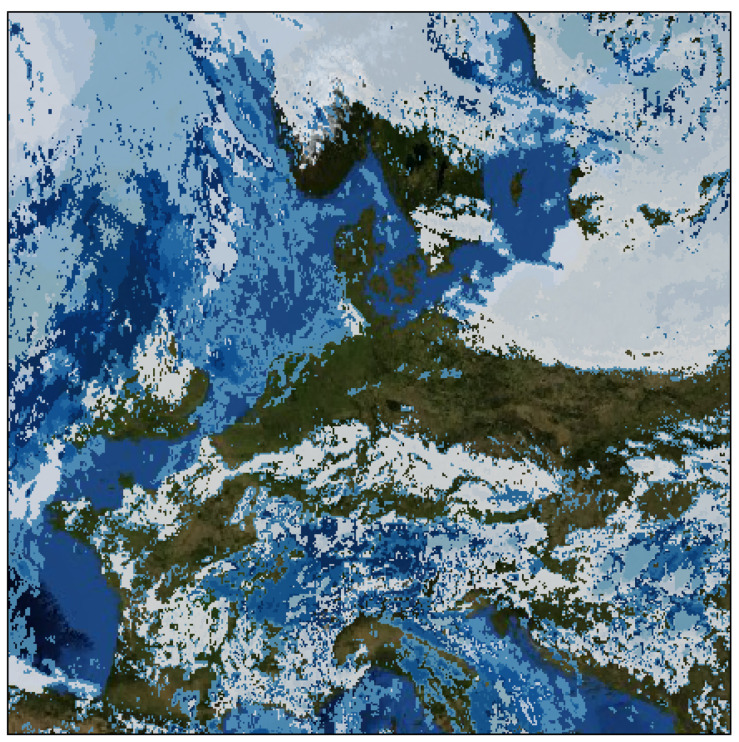
CloudCast sample 728 × 728 pixels. Europe region. Coloured regions correspond to the first four classes.

As suggested by the authors, the first four classes should be eliminated to work with a dataset containing only different types of clouds. In this work, this preprocessing was carried out to obtain a dataset in which the pixels belong to the range of classes between 4 and 14. Figure 6 shows a sample of this dataset.

The CloudCast dataset features a spatial resolution of 928 × 1530 pixels, recorded at 15 min intervals from 2017 to 2018, with each pixel representing an area of 3 × 3 km. Additionally, the authors provide a full-resolution dataset centered and projected over Europe (728 × 728 pixels), enabling the standardized benchmarking of computer vision methods. For smaller-scale experiments and analysis, a downscaled low-resolution dataset of 128 × 128 pixels is available, with each pixel representing an area of 15 × 15 km, significantly reducing the size of the dataset compared to the full version.

To evaluate the performance of the proposed architecture in a real-world scenario, we performed our first experiments using the first $5.2\times 10^{4}$ images of the 128 × 128 resolution CloudCast dataset.

In the tests, we fed the network with $5\times 10^{4}$ training examples, with the first $10^{3}$ examples used for the initial transient phase. Once we calculated the weights of the output layer, we used the next $10^{3}$ examples for testing, calculating the next value at each step. This setup provides a prediction horizon of 15 min. For the final comparison, where we compared the results with other architectures, we used a prediction horizon of 1 h to align our results with those of the compared architectures.

## 5. Results and Discussion

### 5.1. MNIST and FashionMNIST Datasets

In our previous study, we performed a performance comparison between the architectures mentioned. To obtain the mean and standard deviation values, 100 runs of each of the experiments were performed. The results of this comparison are shown in Table 4.

To determine the accuracy of the network, we calculated the ratio of correctly classified examples to the total number of examples in the test phase (see Formula (2)).

(4) $$\mathrm{Accuracy}=\frac{\mathrm{Number}\;\mathrm{of}\;\mathrm{Correct}\;\mathrm{Predictions}}{\mathrm{Total}\;\mathrm{Number}\;\mathrm{of}\;\mathrm{Predictions}}$$

As observed, the MRESN architecture consistently achieves a higher accuracy across most cases and demonstrates competitive execution times. Consequently, for the resource-constrained device benchmark, specifically employing a DPU, we opted to focus comparisons solely between this architecture and the vanilla ESN.

In our experiments, running a vanilla ESN architecture with 8000 nodes on the DPU was not feasible due to memory limitations, resulting in crashes as indicated in Table 5. However, we successfully executed an MRESN network consisting of 16 repositories, each containing 500 nodes, on the same device. Remarkably, despite the reduction in the number of nodes, the multi-repository network demonstrated an improved overall accuracy compared to the vanilla ESN. For further insights into training times and accuracy comparisons between a DPU and CPU, please refer to Table 5.

**Figure 7:**
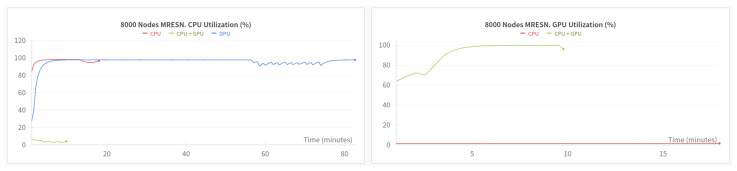
Execution times on CPU vs. GPU vs. DPU for 8 × 1000 nodes MRESN.

One point to bear in mind is that the duration of training is closely related to the device on which it is performed. With low-power devices with limited resources, the time required to train a reservoir-based model on a DPU is much longer than on a CPU or GPU, but provided that the initial conditions are the same, the accuracy performance will be identical in all three cases. Figure 7 shows a comparison in terms of execution time between the same MRESN model that was trained on the DPU, on the CPU and on the CPU + GPU.

### 5.2. CloudCast Dataset

In contrast to the previous datasets, the dataset at hand exhibits a temporal relationship between consecutive images. Therefore, certain adjustments in the architecture are imperative for the network to effectively leverage this information. Specifically, whereas we employed two initial transient steps for each image when working with MNIST and FashionMNIST, we then executed an initial transient of 1000 steps at the onset of training. Subsequently, to compute the new state, we relied on the preceding state of the network instead of the neutral vector previously utilized for MNIST and FashionMNIST.

In contrast to with the previous datasets, where the prediction involved feeding the network a 28 × 28 pixel image and determining its class, our current task entails predicting the class of a particular pixel over time. This prediction reflects the type of cloud present over that pixel at a given time, which is influenced by the evolving classes of nearby pixels. In simpler terms, the network must learn that if a cloud is moving from the west, it will eventually reach the target pixel, unless it dissipates before reaching it. This time, the size of the input will depend directly on the pixel radius we use. As the image is flattened before it is fed into the network, we used Formula (5) to determine the size of the input:

(5) $$\left|u\right|=1\;x\;{\left(R+1\right)}^{2}$$

where *u* is the input, and *R* is the radius used.

**Figure 8:**
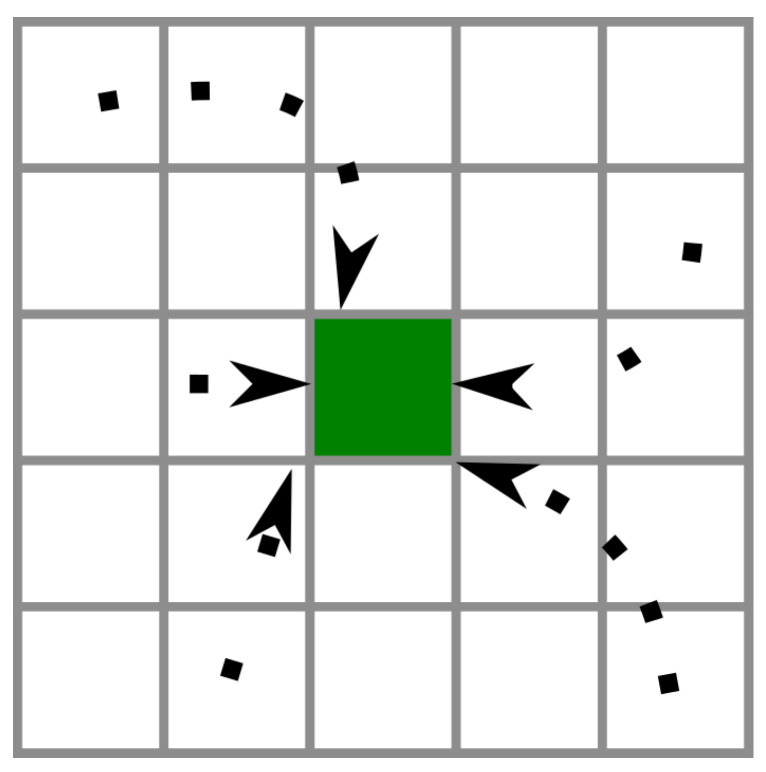
Effect of neighboring pixels on the target pixel over time. The dashed lines represent successive time steps. Certain pixels exert immediate influence on the target (green cell), while others require multiple steps for their information to reach the target.

The first step for CloudCast is to select a target pixel and define a pixel radius around it. The goal is to train the model to detect the influence of surrounding pixels on the target pixel over time intervals. Figure 8 illustrates this concept with a hypothetical target pixel (green cell) and the influence of neighboring pixels within its radius. Dashed lines depict successive time steps, indicating the propagation of information from each pixel to the target. While some pixels exert an immediate influence on the target, others require multiple time steps for their information to reach it.

To study the influence of different hyperparameters on this dataset, we first performed a grid search. Subsequently, we performed a Particle Swarm Optimization (PSO) search to check if the conclusions drawn from the previous search were correct. We can see the range of hyperparameters used for the grid search in Table 6.

**Figure 9:**
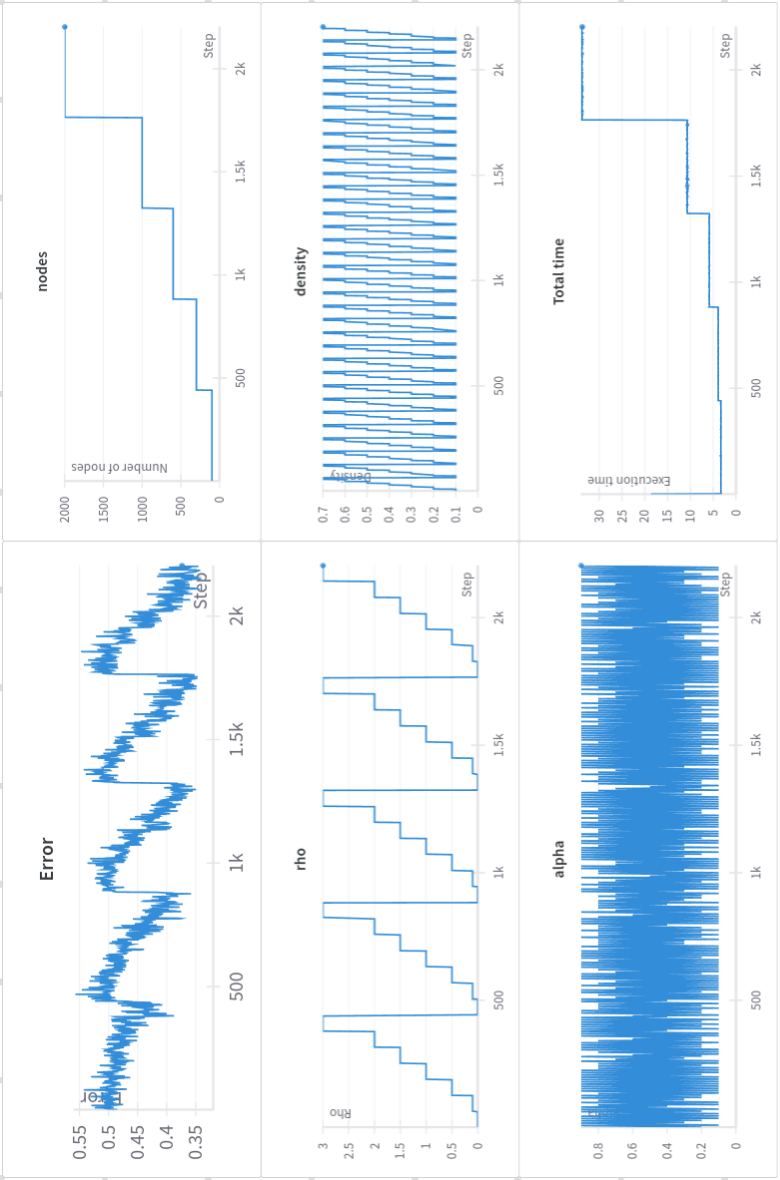
ESN grid search for CloudCast dataset. Big influence of spectral radius on results.

As depicted in Figure 9, the number of nodes in the network exhibits diminished influence on the results compared to previous experiments. While increasing the network size leads to incremental improvements, networks of 500 nodes or more yield similar results, with maximum accuracies hovering around 65%. This behavior can be attributed to the use of 7 × 7 pixel images, which are considerably smaller than the 28 × 28 pixel MNIST images used previously.

Conversely, the spectral radius emerges as the key parameter with a significant impact on the results. A high spectral radius is essential to achieve a satisfactory performance. As already mentioned, the spectral radius and the input scaling take on increased importance in the presence of temporal relationships within the input data, underlining their fundamental roles in this task.

**Figure 10:**
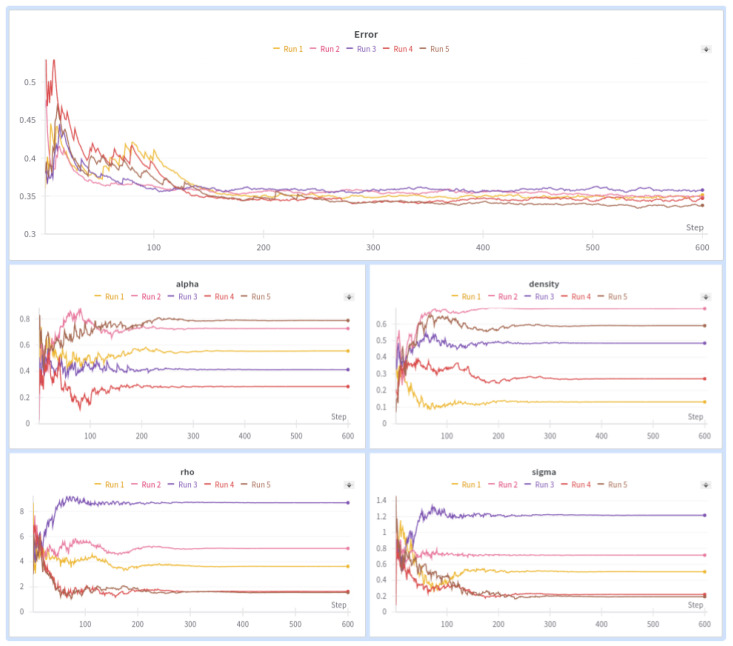
PSO results for ESN architecture.

When conducting a hyperparameter search on the ESN model using the PSO algorithm, we observed that high values of rho, specifically those exceeding 1.5, yielded better results, with average accuracies around 66% and a maximum accuracy close to 70%. The results of the PSO algorithm for the ESN architecture are presented in Figure 10. To enhance visual clarity, only 5 out of the 50 conducted experiments are displayed. It is observed that the randomness in generating the reservoir leads to a rugged hyperparameter space, where different combinations yield similar results. The specific parameters used in the PSO search are detailed in Table 7.

**Figure 11:**
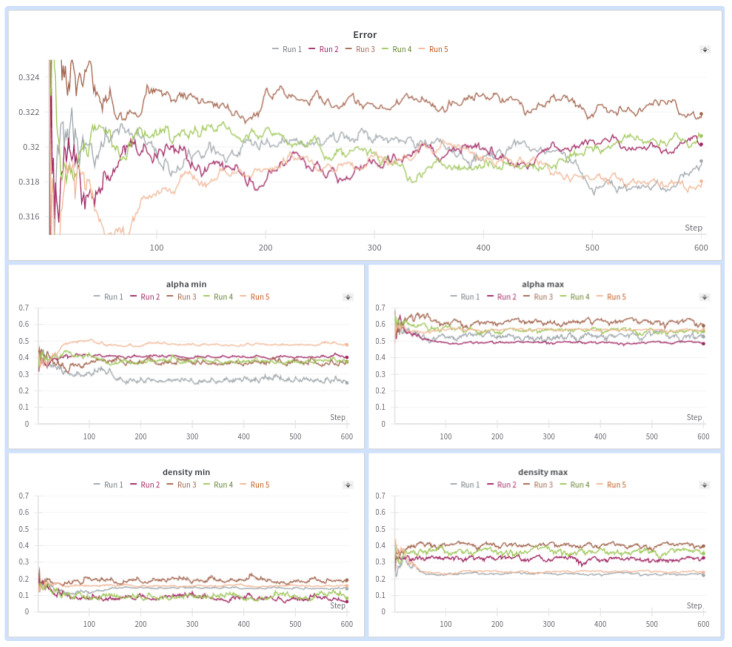
PSO over MRESN architecture.

The hyperparameter search in the MRESN model used the same parameters described in Table 7, but achieving convergence in 30 steps was more challenging due to the greater number of hyperparameters in this model. Nevertheless, the MRESN model outperformed the ESN model, with an average accuracy of around 68% and a maximum accuracy exceeding 70%. Allowing multiple reservoirs to operate independently increases the variety of features that can be processed by the network, which enhances overall performance. The results of the PSO algorithm for the MRESN model are shown in Figure 11. We included only 5 out of the 50 runs to illustrate that, due to the inherent randomness in the reservoirs, different sets of hyperparameters can yield similar results. To improve the figure’s visibility, we smoothed the lines and presented the top five results obtained from the PSO algorithm. These top five results are detailed in Table 8.

In [6], the authors used the CloudCast dataset to test the performances of various models. Since these models are fed exclusively from this dataset and do not use additional information such as humidity and temperature data, which is often included in similar tasks, comparing our models with those of this work provided a fair assessment of the model performance. The tested models were the following:

- Optical Flow Algorithm (TV-L1);
- Autoencoder ConvLSTM (AE-ConvLSTM);
- Multi-Stage Dynamic Generative Adversarial Networks (MD-GAN).

Table 9 compares these models with the MRESN architecture.

Finally, the MRESN models were deployed on resource-constrained devices to assess their feasibility in home applications. For this purpose, the models were installed on a single data processing unit (DPU), a single Raspberry Pi 4, and a single Raspberry Pi 5. To obtain the mean and variance measurements, 100 experiments were conducted on each device. The corresponding run times for each device are detailed in Table 10.

Given the 15 min time resolution of the dataset, the network training times were sufficiently short to allow for retraining with each new input. Even on a resource-constrained device such as the Raspberry Pi 4, a six-reservoir network with 300 nodes can be trained in approximately 13 min. Although 5 × 300 node networks already provide good performance for the CloudCast dataset, we wanted to further increase the number of repositories to see the limit we could reach on the Raspberry Pi 4, as this device has the smallest amount of memory (2 GB). This limit was reached on the 7 × 300 node network, where the experiments failed due to a lack of memory.

## 6. Conclusions

Given the inherent advantages of ESN architectures over CNNs in terms of training time and resource usage, together with the superior efficiency and performance demonstrated by MRESNs over ESNs, the proposed architecture shows promise for deployment in cloud and edge computing environments, especially in specific scenarios. DNNs have traditionally been trained on computers equipped with one or more GPUs, as conducting these computations without GPU acceleration would significantly prolong the training time. In contrast, ESN-based architectures offer the flexibility of training on CPUs, thereby unlocking new opportunities for servers equipped with both GPUs and DPUs. In such configurations, GPUs can be allocated for heavier models, like those based on DNN architectures, while reservoir-based architectures can be parallelly trained on DPUs, which is a direction that we plan to explore in future research. In this setup, the training time required on DPUs becomes less critical, as these devices are designed to handle network traffic and remain consistently active. Consequently, we can leverage their low workload periods to conduct training tasks. Furthermore, given that graphics cards are one of the most energy-intensive components, replacing GPU-intensive models with reservoir-based architectures can lead to significant energy savings.

Despite the extended training times associated with reservoir-based architectures, the low power consumption of DPUs renders them an appealing long-term option. MRESNs offer a good balance between accuracy and computational efficiency, making them an ideal choice for scenarios where both performance and resource constraints are paramount.

Comparative analyses further confirm the superior precision and execution time of the MRESN approach over the original ESN architecture. MRESN attains a higher accuracy while demanding less training time and disk space for the utilized structures. Since reservoir-based architectures, unlike deep neural networks, can be trained on CPUs in an acceptable time, MRESNs can be a good substitute for them in certain scenarios, for example, where network-level processing is required on devices such as DPUs or home stations. While these architectures may not achieve the performance levels of DNNs, they can serve as an initial layer in a multi-tiered processing approach. Here, a model deployed on the DPU could execute initial decision-making processes before routing the input to specialized servers for further processing. Exploring this concept further is among the avenues we intend to investigate in future research.

In this study, we extended our previous work, which explored these architectures on the MNIST and FashionMNIST datasets, by applying the network to a task characterized by both spatial and temporal relationships: the CloudCast dataset. For this dataset, it was necessary to adapt our architecture to determine the class to which a given pixel belongs over time. As previously demonstrated with MNIST, the ESN achieved a maximum performance of approximately 70%, whereas the MRESN-based architecture surpassed this threshold, reaching accuracy peaks close to 73%.

By leveraging the dataset’s inherent temporal relationships, MRESNs demonstrated robust performance, surpassing conventional ESN models in terms of accuracy and efficiency. Furthermore, our experiments underscored the critical role of the spectral radius in determining the performance of reservoir-based architectures, particularly in scenarios with temporal dependencies.

Through an evaluation of the runtime performance on diverse low-resource devices, including platforms like BlueField 2.0 and Raspberry Pi, we showcased the practical versatility of MRESNs in various computing scenarios, spanning from cloud and edge computing to home applications.

In summary, our results highlight the versatility and effectiveness of the MRESN architecture in addressing the challenges posed by datasets with spatial and temporal dependencies, such as the CloudCast dataset. By achieving superior performances while maintaining computational efficiency, MRESNs emerge as a promising solution for deployments where both accuracy and resource constraints are paramount. These results extend the reach of reservoir-based architectures and highlight their potential for a wide range of applications in image processing and beyond.

## Figures and Tables

**Table 1:** Number of trainable parameters in DNN architectures.

Model/Architecture	Trainable Parameters
LeNet-5	≈70,000
AlexNet	≈60 Millions
VGGNet-16	≈140 Millions
ResNet50	≈25 Millions
GoogLeNet	≈15 Millions

**Table 2:** Overview of previous work: scenarios of different ESN-based architectures.

Work	Architecture	Target	Context	Metric
[9]	ESN	Classification	Audio signal	WER
[7]	ESN	Prediction	Word Seq.	Cosine, AUC
[8]	ESN	Classification	Word seq.	Error rate
[13]	ESN	Prediction	Time series	NMSE
[11]	ESN	Classification	Image	Error rate
[14]	ESN	Prediction	Time series	RMSE
[12]	ESN	Prediction	Vector	AUC
[20]	ESN + MLP	Segmentation	Image	Accuracy
[21]	ESN + LSTM	Prediction	Time series	RMSE, MAE
[22]	ESN + CNN	Prediction	Time series	MAE, MAPE
[15]	ESN	Classification	Image	Error rate
[17]	RCN	Image recognition	Image/Video	Error rate
[16]	ESN	Predict./Classif.	Various	Various
[18]	ESN + CNN	Classification	Images	Error rate
[19]	ESN	Classification	Images	Error rate

**Table 3:** Characteristics of the devices used in this work.

	BlueField 2.0	Raspberry Pi 4	Raspberry Pi 5	Server
**CPU**	ARMv8 A72	ARMv8 A72	ARM Cortex A76	AMD Ryzen 7 5800X
**Cores**	8	4	4	16
**GPU**	-	-	-	Nvidia GeForce RTX 4090
**RAM**	16 GB	2 GB	8 GB	64 GB
**OS**	Ubuntu 20.04	Raspberry OS	Raspberry OS	Ubuntu 20.04
**kW/h**	6.4	5	5	-

**Table 4:** Global execution time. The number of reservoirs is expressed by *r*, and *N* represents the number of nodes per reservoir. Best results have been highlighted for easy of reading.

Architecture	*r × N*	Time CPU	Time GPU	Accuracy MNIST	Accuracy FashionMnist
ESN	1 × 20,000	04:12:00	00:40:59	97.70 ± 0.10	88.70 ± 0.10
MRESN	20 × 1000	**00:39:59**	00:11:30	**98.36 ± 0.07**	**89.13 ± 0.09**
deepESN	10 × 2000	01:02:03	**00:10:53**	89.97 ± 0.011	69.20 ± 0.016
deepESN-IA	10 × 2000	01:29:55	00:12:29	96.63 ± 0.003	86.43 ± 0.003
ESN	1 × 15000	02:16:00	00:20:42	97.55 ± 0.04	88.48 ± 0.06
MRESN	15 × 1000	**00:26:06**	00:07:30	**98.28 ± 0.03**	**89.10 ± 0.20**
deepESN	8 × 2000	00:46:29	**00:07:29**	89.46 ± 0.013	68.03 ± 0.014
deepESN-IA	8 × 2000	01:09:03	00:08:39	96.78 ± 0.002	86.97 ± 0.003
ESN	1 × 10,000	01:11:00	00:08:36	97.28 ± 0.15	88.36 ± 0.13
MRESN	10 × 1000	**00:18:30**	00:04:00	**97.76 ± 0.30**	**88.45 ± 0.20**
deepESN	5 × 2000	00:26:48	**00:03:32**	90.54 ± 0.014	69.42 ± 0.021
deepESN-IA	5 × 2000	00:39:58	00:04:09	97.07 ± 0.002	87.23 ± 0.003
ESN	1 × 6000	00:31:30	00:02:24	96.80 ± 0.10	**87.84 ± 0.15**
MRESN	6 × 1000	00:16:12	00:02:01	**97.12 ± 0.04**	87.50 ± 0.15
deepESN	3 × 2000	**00:14:26**	**00:01:40**	90.86 ± 0.009	69.58 ± 0.016
deepESN-IA	3 × 2000	00:21:54	00:02:03	97.01 ± 0.001	87.37 ± 0.003
ESN	1 × 2000	**00:01:30**	**00:00:31**	95.00 ± 0.14	86.44 ± 0.30
MRESN	4 × 500	00:01:45	00:00:51	**96.64 ± 0.001**	**86.77 ± 0.13**
deepESN	2 × 1000	00:01:36	00:01:12	83.95 ± 0.02	63.16 ± 0.02
deepESN-IA	2 × 1000	00:02:17	00:01:14	95.99 ± 0.003	82.59 ± 0.001

**Table 5:** DPU results. The number of reservoirs is expressed by *r*, and *N* represents the number of nodes per reservoir.

Architecture	*r × N*	Time	Accuracy	
			MNIST	FashionMNIST
ESN	1 × 8000	crashed	crashed	crashed
MRESN	16 × 500	79.9 min.	97.20 ±0.07	88.48 ±0.09
ESN	1 × 6000	157 min.	96.80 ±0.10	87.84 ±0.15
MRESN	12 × 500	61.0 min.	97.05 ±0.04	87.5 ±0.15
ESN	1 × 4000	72.5 min	96.03 ±0.11	87.36 ±0.11
MRESN	8 × 500	36.7 min	96.40 ±0.06	87.45 ±0.12
ESN	1 × 2000	22.2 min.	95.00 ±0.14	86.44 ±0.30
MRESN	4 × 500	18.1 min.	95.60 ±0.15	86.77 ±0.13

**Table 6:** Range of hyperparameter values for grid search in vanilla ESN for CloudCast dataset. N is used to express the number of nodes in the network, and D is used to express the density of the reservoir.

Parameter	Minimum Value	Maximum Value
N	$1\times 10^{2}$	$2\times 10^{3}$
D	0.1	0.7
α	0.1	0.9
ρ	0.001	3.0
***R***	3	
***Initial transient***	$1\times 10^{3}$	
***Train steps***	$5\times 10^{4}$	
***Test steps***	$1\times 10^{3}$	
β	$10^{-8}$	

**Table 7:** PSO parameters for ESN and MRESN models.

	Population	Iterations	C1	C2	w
**ESN**	20	30	1.5	1.2	0.5
**MRESN**	20	30	1.5	1.2	0.5

**Table 8:** Top five results from the PSO hyperparameter search in the MRESN model.

	1°	2°	3°	4°	5°
**Error**	0.274	0.281	0.286	0.286	0.287
***r × N***	5 × 300	5 × 300	3 × 300	5 × 300	5 × 300

**Table 9:** Comparison of model performances.

Model	AE-ConvLSTM	MD-GAN	TV-L1	MRESN 5 × 300
**Accuracy**	73.58%	73.32%	70.07%	70.58%

**Table 10:** Execution time (seconds) for different low-resource devices.

*r × N*	BlueField 2.0	Raspberry Pi 4	Raspberry Pi 5
2 × 300	85.8 ± 0.78	187.56 ± 13.13	31.96 ± 4.61
3 × 300	71.7 ± 0.65	298.74 ± 70.54	49.89 ± 2.64
4 × 300	95.64 ± 1.56	419.65 ± 82.54	77.94 ± 3.49
5 × 300	131.61 ± 0.88	572.46 ± 51.98	107.03 ± 2.68
6 × 300	169.4 ± 0.67	783.19 ± 50.55	140.21 ± 1.62
7 × 300	210.2 ± 0.85	Crashed	179.44 ± 1.73

## Data Availability

The datasets used to train and evaluate these networks are publicly available at https://git-disl.github.io/GTDLBench/datasets/mnist_datasets/ (accessed on accessed on 18 April 2024), https://www.kaggle.com/datasets/zalando-research/fashionmnist (accessed on 18 April 2024), and https://vision.eng.au.dk/cloudcast-dataset/ (accessed on 18 April 2024).

## Abbreviations

The following abbreviations are used in this manuscript:

CNN	Convolutional Neural Network;
DNN	Deep Neural Network;
DPU	Data Processing Unit;
ESN	Echo State Network;
IoT	Internet of Things;
LSTM	Long Short-Term Memory;
MLP	Multi-Layer Perceptron;
MRESN	Multi-Reservoir Echo State Network;
NIC	Network Interface Card;
PSO	Particle Swarm Optimization;
RC	Reservoir Computing;
SaaS	Software as a Service.
